# Auditory dominance in motor-sensory temporal recalibration

**DOI:** 10.1007/s00221-015-4497-0

**Published:** 2015-11-26

**Authors:** Yoshimori Sugano, Mirjam Keetels, Jean Vroomen

**Affiliations:** Department of Industrial Management, Kyushu Sangyo University, 3-1 Matsukadai, 2-Chome, Higashi-ku, Fukuoka, 813-8503 Japan; Department of Cognitive Neuropsychology, Tilburg University, P.O. Box 90153, 5000 LE Tilburg, The Netherlands

**Keywords:** Temporal recalibration, Motor-visual, Motor-auditory, Tapping, Perception of synchrony, Multisensory processing

## Abstract

Perception of synchrony between one’s own action (e.g. a finger tap) and the sensory feedback thereof (e.g. a flash or click) can be shifted after exposure to an induced delay (temporal recalibration effect, TRE). It remains elusive, however, whether the same mechanism underlies motor-visual (MV) and motor-auditory (MA) TRE. We examined this by measuring crosstalk between MV- and MA-delayed feedbacks. During an exposure phase, participants pressed a mouse at a constant pace while receiving visual or auditory feedback that was either delayed (+150 ms) or subjectively synchronous (+50 ms). During a post-test, participants then tried to tap in sync with visual or auditory pacers. TRE manifested itself as a compensatory shift in the tap–pacer asynchrony (a larger anticipation error after exposure to delayed feedback). In experiment 1, MA and MV feedback were either both synchronous (MV-sync and MA-sync) or both delayed (MV-delay and MA-delay), whereas in experiment 2, different delays were mixed across alternating trials (MV-sync and MA-delay or MV-delay and MA-sync). Exposure to consistent delays induced equally large TREs for auditory and visual pacers with similar build-up courses. However, with mixed delays, we found that synchronized sounds erased MV-TRE, but synchronized flashes did not erase MA-TRE. These results suggest that similar mechanisms underlie MA- and MV-TRE, but that auditory feedback is more potent than visual feedback to induce a rearrangement of motor-sensory timing.

## Introduction

Perception of temporal synchrony between one’s own action (e.g. finger tapping) and sensory feedback thereof (e.g. tactile, visual, and auditory signals) is quite flexible. Their subjective relative timing can be easily shifted after exposure to an artificially induced temporal delay of the sensory feedback (Stetson et al. 2006; Heron et al. 2009; Sugano et al. 2010, 2012, 2014; Stekelenburg et al. 2011; Arnold et al. 2012; Keetels and Vroomen 2012; Parsons et al. 2013; Rohde and Ernst 2012; Rohde et al. 2013, 2014b; Toida et al. 2014a, b; Tsujita and Ichikawa 2012; Vercillo et al. 2014; Yarrow et al. 2013). This is usually referred to as motor-sensory temporal recalibration because the shift is, presumably, induced to reduce the timing error between events that normally occur together.

**Fig. 1:**
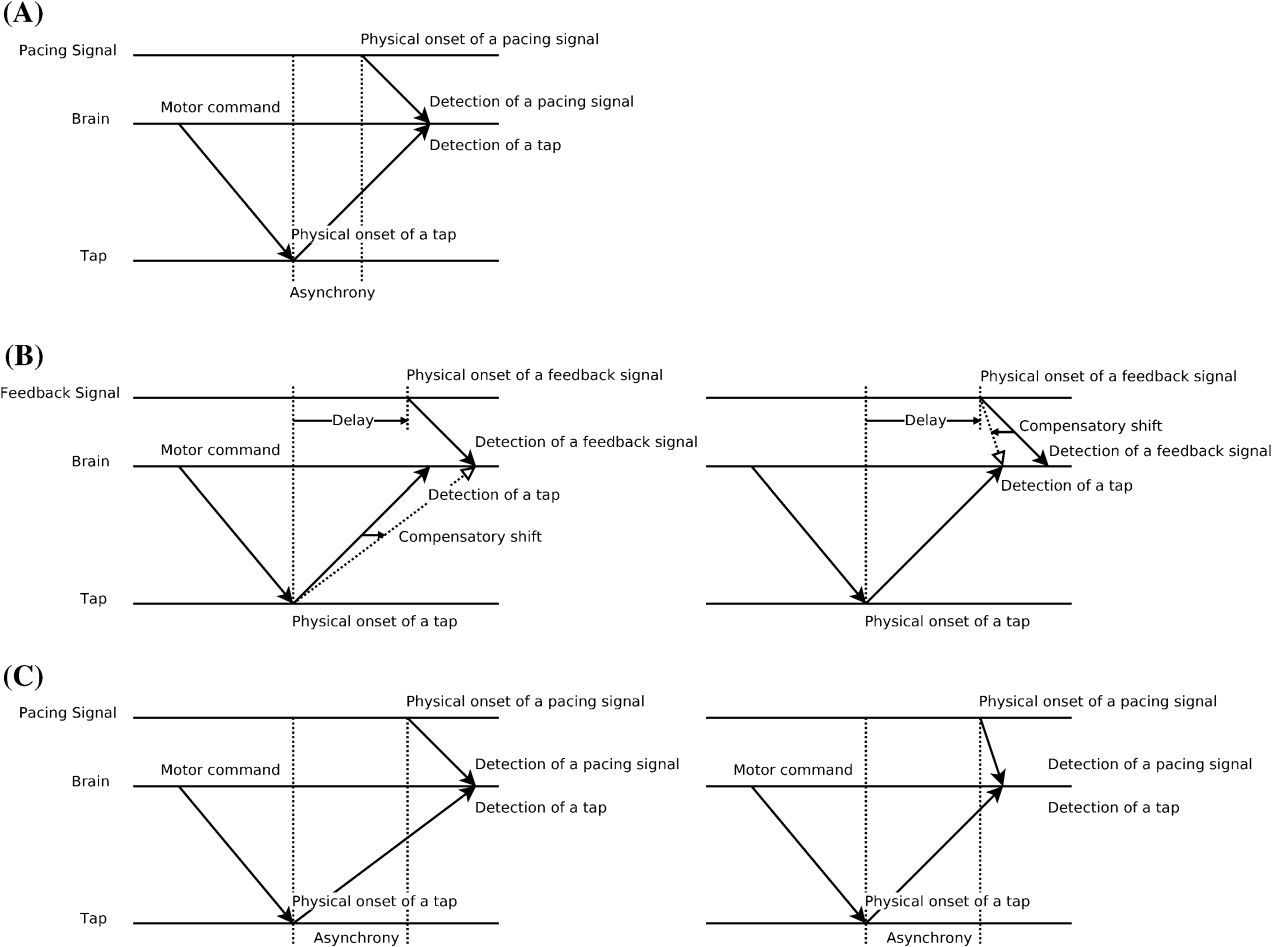
Schematic illustration of the compensatory shift in the tap-asynchrony (i.e. an increase in anticipation error) after exposure to delayed feedback. Figure is taken from Sugano, Keetels and Vroomen (2012) that is modified from Aschersleben and Prinz (1997)’s Figure 1. **a** Synchronous tapping before adaptation: the latency difference between detection of a pacing signal (sound or flash) and tap gives rise to a tap-asynchrony (tap-before-pacing-signal; Paillard–Fraisse hypothesis, Paillard 1949; Fraisse 1980; Aschersleben and Prinz 1997). **b** Exposure to delayed feedback: to realign the delayed external feedback after a voluntary tap, participants shift their representation of when the pad was touched (*left panel*) or when the pacing signal came (*right panel*), thus causing adaptation. **c** Synchronous tapping after adaptation: taps are given earlier due to the lingering effect of adaptation to a delay that either slowed down the detection of a tap (*left panel*) or sped up the detection of the pacing signal (*right panel*)

The shift in relative timing has traditionally been measured via simultaneity judgments (SJs) or temporal order judgments (TOJs). However, recently we demonstrated that tapping in synchrony with an external pacing signal (an ST task for “synchronous tapping”) also provides a viable measure of temporal recalibration (Sugano et al. 2012) that, arguably, is easier and more natural than SJ or TOJ. Figure 1 shows a schematic illustration of how sensory-specific timing and/or movement-related timing (motor/tactile) shifts in an ST task after participants are exposed to delayed feedback. The latency difference between detection of a pacing signal (a sound or a flash) and a finger tap gives rise to a tap-asynchrony (Fig. 1a: “tap-before-pacing-signal”; Paillard–Fraisse hypothesis, Paillard 1949; Fraisse 1980; Aschersleben and Prinz 1997). Figure 1b shows how adaptation to a delayed sensory feedback occurs in order to compensate the delay: the shift might be related to motor timing (“when did I touch the surface?”, or “when did I move my finger?”) or timing of the sensory feedback (“when did I hear the sound or see the flash?”). Figure 1c shows how timing is modified after adaptation to a delay if motor timing (left panel) or timing of the sensory feedback shifts (right panel). In both cases, one expects the tap-asynchrony to *increase* (i.e. a larger anticipation error) after observers are adapted to a delay.

At present, many researchers have demonstrated motor-sensory recalibration (Stetson et al. 2006; Heron et al. 2009; Sugano et al. 2010, 2012, 2014; Stekelenburg et al. 2011; Arnold et al. 2012; Keetels and Vroomen 2012; Parsons et al. 2013; Rohde and Ernst 2012; Rohde et al. 2013, 2014a, b; Timm et al. 2013; Toida et al. 2014a, b; Tsujita and Ichikawa 2012; Vercillo et al. 2014; Watanabe et al. 2011; Yamamoto and Kawabata 2011, 2014; Yarrow et al. 2013). However, it is still unclear which component is shifted in motor-sensory temporal recalibration, motor or sensory, and it is still in debate whether motor-sensory temporal recalibration is identical across sensory parings. Some researchers have shown that motor-sensory temporal recalibration transfers to other sensory modalities using TOJs (Heron et al. 2009; Sugano et al. 2010). They argued that the same mechanism underlies temporal recalibration across these sensory parings. However, others have shown that there might be sensory-specific shifts as well (Stekelenburg et al. 2011; Sugano et al. 2012; Toida et al. 2014b). For example, Stekelenburg et al. (2011) demonstrated that motor-visual (MV) temporal recalibration might occur at early processing stages because the visual P1 in the EEG signal (at ~85–~150 ms) was attenuated after adaptation to delayed visual feedback. Likewise, Toida et al. (2014b) argued for early involvement because the auditory-evoked P2 was increased after motor-auditory (MA) temporal recalibration. Sugano et al. (2012) tested whether temporal recalibration transferred between the MA and MV domains, and showed an asymmetric transfer in which MV transferred to MA but not in the reverse direction, suggesting a sensory-specific component in motor-sensory temporal recalibration.

### Purpose of the study

The present study aimed to further elucidate whether MA and MV temporal recalibrations share similar mechanisms. For that purpose, we adopted an exposure-test paradigm in which participants were exposed, across trials, to visual and auditory feedback that was either synchronous or delayed relative to voluntary movements of finger taps. This design allowed us to examine whether there is crosstalk between modalities. As mentioned above, recalibration can be achieved by either a shift of sensory-specific timing and/or movement-related timing. By measuring crosstalk between modalities, one can evaluate to what extent they are mediated by an amodal mechanism. If MA and MV temporal recalibrations are caused by the same amodal mechanism, one expects that the *size* and *build*-*up* of them will be the same as there is complete crosstalk. Alternatively, if separate mechanisms are involved, there might be differences in either the *size* and/or the *build*-*up* of the TRE (tested in experiment 1) (see Sugano et al. 2012). Furthermore, if the same amodal mechanism underlies MA and MV temporal recalibrations, one expects that exposure to synchronized feedback will erase temporal recalibration across modalities. In contrast, one expects modality-specific effects to survive even after mixing sync and delay if sensory-specific components are involved (tested in experiment 2).

Perception of MA and MV timing was examined in a tapping task in which participants tried to sync their finger taps with auditory or visual pacing signals (Sugano et al. 2012, 2014; Rohde et al. 2014b). This ST task provides an implicit measure of relative timing that does not contain overt estimates of timing or duration, but still engages a strict timing mechanism (Coull and Nobre 2008). Therefore, it is thought to be free from cognitive biases that the TOJ and SJ tasks usually have and it can therefore be used as a viable measure of temporal recalibration (Sugano et al. 2012, 2014).

## Experiment 1: Exposure to consistent MA- or MV-delays

### Methods

#### Participants

Twenty-seven students from Kyushu Sangyo University participated in the experiment (three were female, mean age was 21.8, and one was left-handed but used a computer mouse by the right hand). About half of them (thirteen) were assigned to the synchronous feedback condition in which the sensory feedback of MA and MV was always subjectively synchronous (50 ms). The remaining half (fourteen) were assigned to the delayed feedback condition, in which the sensory feedback of MA and MV was always somewhat delayed (150 ms). These values were chosen because they yielded consistent TREs in previous reports (Sugano et al. 2012, 2014).^1^ All participants had normal hearing and normal or corrected-to-normal vision. Written informed consent was obtained from each participant. The experiment was approved by the Local Ethics Committee of Kyushu Sangyo University and followed the Declaration of Helsinki.

#### Stimuli and apparatus

Participants sat at a desk in a dimly lit booth looking at a 17-inch CRT monitor (Fujitsu FMV-DP97W3G running with 100 Hz refresh rate) at approximately 60 cm viewing distance. The visual stimulus was a 1-cm white square (30 ms duration, 9 cd/m^2^) with a black background (0 cd/m^2^) on the CRT monitor. The auditory stimulus was a 2000-Hz pure tone pip (30 ms duration with 2 ms rise/fall slope) presented via headphones (Sony MDR-Z900) at 74 dB(A). A 1-cm red square (30 ms duration, 3 cd/m^2^) and a 2250-Hz pure tone pip [30 ms duration with 2 ms rise/fall slope, 74 dB(A)] were used for catch trials (see “Design and procedure”). White noise was continuously presented via headphones at 68 dB(A) to mask faint sound of mouse-presses. A special gaming mouse (Logitech G300) was used to obtain high temporal resolution (2-ms polling interval). Stimulus presentation and response detection were controlled by E-prime software running on a general PC/AT personal computer (Compaq EVO D300). The temporal resolution of stimulus control and response detection was ~ 1 ms as verified by a multiple-trace oscilloscope.

#### Design and procedure

Test type (pre- vs. post-test) and exposure modality (visual vs. auditory) were within-subject factors, while exposure delay (50 vs. 150 ms) was a between-subjects factor to avoid crosstalk between exposure delays.

**Fig. 2:**
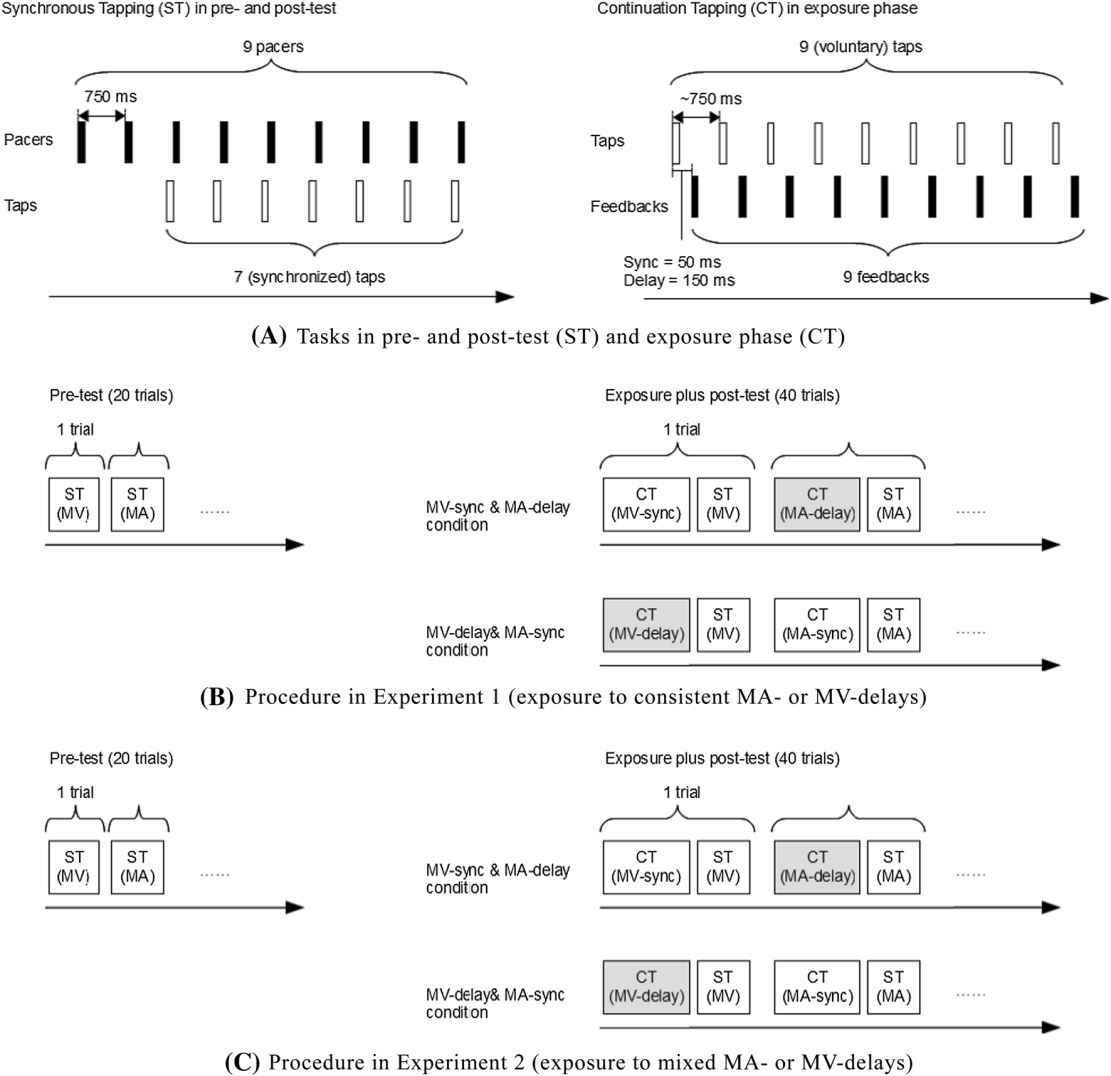
Schematic illustration of **a** the tasks and the experimental procedure in **b** experiment 1 and in **c** experiment 2. Visual and auditory trials were alternated in a pseudo-random fashion across trials. Within a trial, the pacer in the ST task and the feedback in the CT task were presented via the same modality. In experiment 1, each participant experienced either a delayed or a non-delayed feedback (delays were consistent), which were presented via different sensory modalities. In experiment 2, each participant experienced both delayed and synchronous feedbacks in the exposure phase. The delays alternated across consecutive trials and were presented in different modalities

The experimental procedure is shown in Fig. 2. We adopted an exposure-test paradigm that consisted of a pre-test, followed by a (mini-)exposure phase and a post-test. The mini-exposure phase and the post-test were repeated many times so that the build-up of adaptation could be measured. In the pre-test, participants pressed a mouse in synchrony with a pacer (a flash on a CRT display or a tone via headphones) with their right hand. The pacers were delivered 9 times at a constant inter-stimulus interval (ISI) of 750 ms. Participants skipped the first two pacing signals to get into the rhythm and then tried to sync their mouse-presses with the following 7 pacers. The pre-test contained 20 trials, 10 with visual pacers and 10 with auditory pacers. They were presented in pseudo-alternating order.

After completion of the pre-test, the exposure/post-test trials began. During exposure, participants made 9 voluntary finger taps (no pacer) trying to maintain an inter-tap interval of ~750 ms. Each tap was followed by a flash or tone (the same stimulus as in ST task) at either 50 ms (synchronous) or 150 ms (delayed), depending on condition. The modality of the feedback signal (visual or auditory) was kept constant within a trial, but alternated in a pseudo-random fashion across trials. At the end of the exposure phase, participants were asked whether a deviant feedback signal (a red flash or a high tone) was presented (a catch trial) or not to make sure that they attended the feedback signal. The post-test immediately followed the exposure phase and was the same as the pre-test in which participants tapped in sync with a pacer. The modality of the pacer matched the modality of the feedback signal of the previous exposure phase (auditory pacers after MA-exposure and visual pacers after MV-exposure). There were 40 exposure/post-test trials, 20 visual and 20 auditory, all presented in pseudo-alternating order. Testing lasted about 1 h including instructions and practice.

### Results

Trials from the practice session were excluded from further analysis. The tap-asynchrony during the synchronous tapping task was defined as the timing differences between the tap and the pacer. It was negative if the tap preceded the pacer. Missing responses and abnormal tap-asynchronies (out of range from −375 to 375 ms, which was a midpoint of the ISI of the pacer) were eliminated from analysis (0.5 % of the total number of taps). Individual tap-asynchronies that were out of range within the mean plus/minus two standard deviations from each participant’s average for each modality were treated as outliers by a per-participant basis and were also eliminated from the analysis. The rest of the tap-asynchronies were analysed and were averaged over trials for each experimental condition.

#### Mean tap-asynchronies

Table 1 shows the mean tap-asynchronies during the tapping task for each condition before (pre-test) and after (post-test) exposure to delayed feedback. The temporal recalibration effect (TRE) was defined as the change in tap-asynchrony from pre- to post-test. If the TRE is negative, it means that the anticipation tendency became greater after exposure to delayed feedback (as expected). The mean TREs for each condition are also shown in Table 1.

**Table 1:** Mean tap-asynchrony and TRE (in milliseconds) per condition in experiment 1 and experiment 2

Experiment	Group	Exposure delay (ms)	Exposure modality	Pre-test	Post-test	TRE (post–pre)	TRE diff per modality (150–50)
Exp 1	MV-sync and MA-sync group (N = 13)	50	MV	−50.1 (10.5)	−58.5 (12.9)	−8.4 (9.9)	
		50	MA	−88.1 (7.0)	−94.3 (6.9)	−6.2 (4.7)	
	MV-delay and MA-delay group (N = 14)	150	MV	−42.2 (10.2)	−77.6 (11.1)	−35.4*** (5.4)	−27.0*
		150	MA	−80.8 (8.1)	−118.8 (7.7)	−37.9*** (4.2)	−31.7***
Exp 2	MV-sync and MA-delay group (N = 14)	50	MV	−56.9 (10.2)	−71.5 (9.7)	−14.6** (5.7)	
		150	MA	−87.7 (7.2)	−115.3 (6.7)	−27.6** (7.6)	−19.5*
	MV-delay and MA-sync group (N = 14)	150	MV	−57.2 (10.6)	−63.7 (10.5)	−6.4 (5.1)	8.2
		50	MA	−96.3 (8.3)	−104.4 (7.6)	−8.1* (3.9)	

Standard errors of mean (SEMs) are shown in parentheses. Negative numbers indicate that a tap precedes a pacer

* *p* < 0.05, one-tailed. ** *p* < 0.01, one-tailed. *** *p* < 0.001, one-tailed

The TREs were entered into a mixed-model ANOVA with exposure delay (lag-50 vs. lag-150 ms) as a between-subjects factor and exposure modality (visual vs. auditory) as a within-subject factor. The ANOVA showed that the main effect of the exposure delay was significant, *F*(1, 25) = 16.1, *p* < 0.001. The main effect of the exposure modality and the interaction between two factors did not reach significance, both *p*’s > 0.05. These results thus indicate that the tap-asynchrony was greater after exposure to delayed feedback (mean TRE = −36.7 ms) than synchronous feedback (mean TRE = −7.3 ms), irrespective of the sensory modality.

In order to test whether there were significant TREs for each condition, one-sample *t* tests were done to see whether the TREs were significantly different from zero. One-sample *t* tests (one sided as there was a clear prediction) on the TRE for each condition showed that both the MV-TRE (−35.4 ms) and the MA-TRE (−37.9 ms) in the MV-delay and MA-delay condition were significantly less than zero, *t*(13) = 6.6 and *t*(13) = 9.1, respectively, both *p*’s < 0.001, whereas the MV-TRE (−8.4 ms) and the MA-TRE (−6.2 ms) in the MV-sync and MA-sync condition were not significantly different from zero, *t*(12) = 0.8, *p* = 0.206, and *t*(12) = 1.3, *p* = 0.104, respectively. If the TRE differences between delay and sync are expressed in the proportion to that delay, they are 27.0 % for the MV-TRE and 31.7 % for the MA-TRE. This is shown as the “TRE diff per modality” in Table 1. Thus, delayed auditory and visual feedback induced equally large TREs to visual and auditory pacers, whereas synchronous feedback did not elicit a TRE.

#### Build-up of TRE

**Fig. 3:**
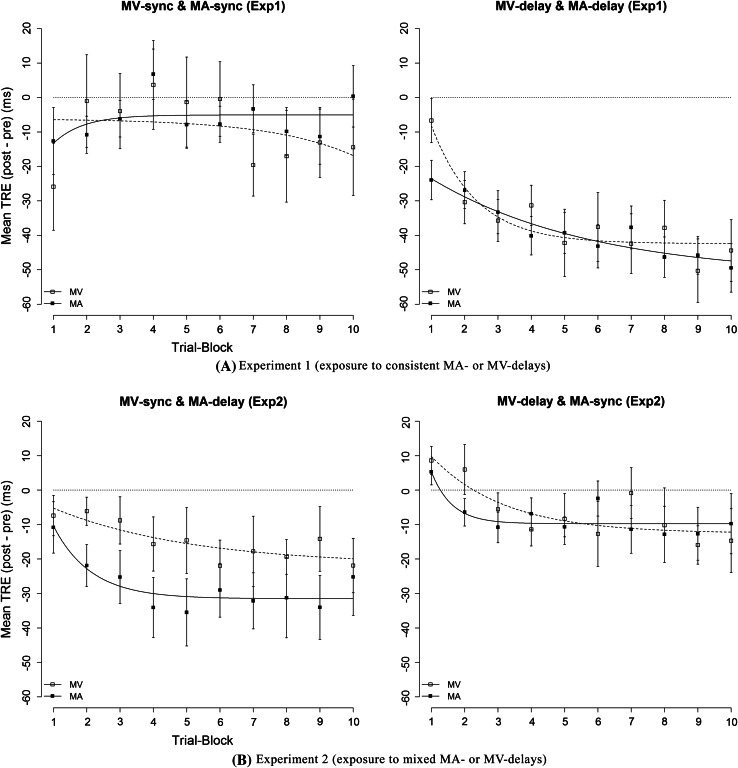
Build-up course of the mean TRE (the difference of tap-asynchrony from pre- to post-test) across trial blocks in **a** experiment 1 and in **b** experiment 2. *Dotted lines* with *white square* marks represent the visual TRE, and *solid lines with black square* marks represent the auditory TRE. One trial block contains 4 adjacent trials (2 visual and 2 auditory). *Error bar* represents a standard error of mean (SEM). An exponential decay function, p0 + p2 × (1 − exp(−p1 × x)), was fitted to the mean TRE per trial block

Although the MV-TRE and the MA-TRE are shown to be equal in size, it does not necessarily mean that their internal mechanisms are same. For example, if their build-up were different, it might also imply that different mechanisms are involved. To examine how the TRE built up across trials, mean TREs per trial block were calculated by subtracting the mean tap-asynchrony in the pre-test from tap-asynchronies for each trial block in the post-test. One trial block consisted of 4 adjacent trials (2 visual and 2 auditory trials). Figure 3 shows how the TREs built up across trial blocks. A nonlinear exponential decay function, p0 + p2 × (1 − exp(−p1 × *x*)), was fitted to the mean TRE per trial block, where p0 reflects the “initial TRE” of adaptation at the first trial block (*x* = 0), p1 reflects the “recalibration rate” (the greater, the faster the decay), and p2 reflects the “max span of TRE”, which is the difference between the “initial TRE” and “final TRE” after adaptation was completed (*x* → *∞*). The fitting was carried out using the NLS function in the statistical package R version 3.1.0 (R Core Team 2014) with the NL2SOL algorithm, which gave the nonlinear least squares estimates of fitting parameters. The fitted lines are also shown in Fig. 3. As can be seen, the MV-TRE and the MA-TRE built up in a very similar way and had not yet reached plateau even at the end of the experiment (20 exposure trials = ~2-min exposure for each modality).

**Fig. 4:**
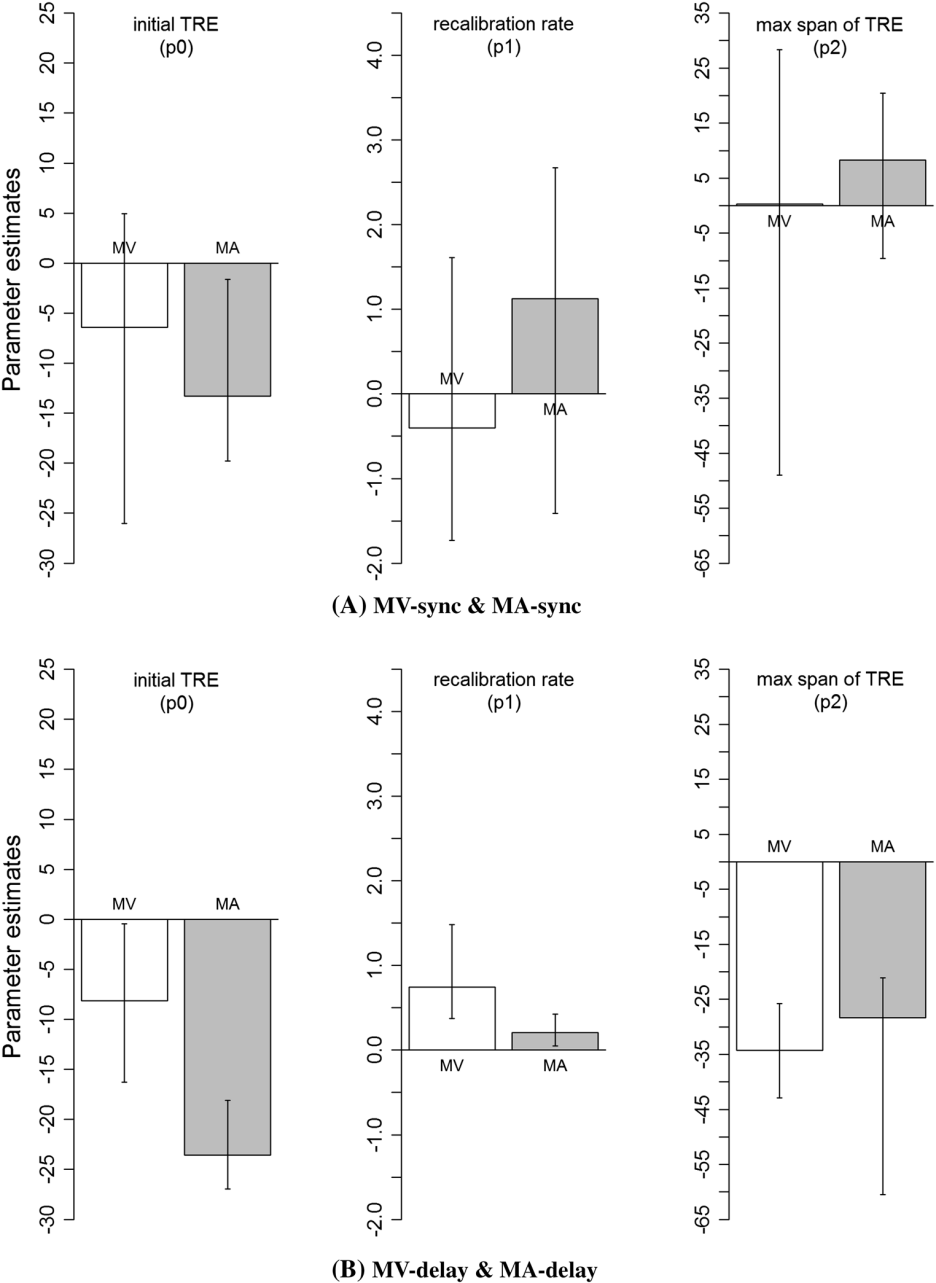
Estimated values of the parameters (p0, p1, and p2) in the nonlinear fitting for the build-up of the mean TRE in experiment 1 (exposure to consistent MV- or MA-delays): **a** MV-sync and MA-sync and **b** MV-delay and MA-delay. *Error bar* represents 95 % bootstrap confidence interval after 2000 simulations

To verify their similarity of build-up more formally, we used a bootstrap method to calculate a confidence interval of each parameter (Efron and Tibshirani 1986; Motulsky and Christopoulos 2003). Figure 4 shows estimated values of the parameters and 95 % bootstrap confidence intervals (CI) after 2000 simulations. As shown in Fig. 4, all the 95 % CIs overlapped between MV and MA, except for the “initial TRE” (p0) and “recalibration rate” (p1) under the MV-delay and MA-delay condition (Fig. 4b), showing that the MV-TRE was initially somewhat smaller (p0 = −8.1), but built up faster (p1 = 0.744) than the MA-TRE (p0 = −23.6, p1 = 0.206).

#### Variability of tap-asynchronies

Some researchers have suggested that a lowered sensitivity to delays is the first stage of temporal recalibration (Navarra et al. 2005; Winter et al. 2008). This might be reflected in an increase in tapping variability before subjective simultaneity shifts. To examine whether the TRE emerged with or without a change in variability, we examined whether variability increased after exposure to delayed feedback. We calculated a mean within-trial standard deviation (WSD) of tap-asynchrony as a measure for tapping variability. The mean WSD is the averaged standard deviation of tap-asynchrony within a trial of 7 individual taps. The group-averaged WSDs are shown in Table 2. We analysed ΔWSD as the difference of the mean WSD from the pre-test to the post-test as an indicator of whether variability increased or not. The mean ΔWSDs for each experimental condition are also shown in Table 2.

**Table 2:** Mean within-trial standard deviation (WSD) of tap-asynchrony and ΔWSD (in milliseconds) per condition in experiment 1 and experiment 2

Experiment	Group	Exposure delay (ms)	Exposure modality	Pre-test	Post-test	ΔWSD (post–pre)	ΔWSD diff per modality (150–50)
Exp 1	MV-sync and MA-sync group (N = 13)	50	MV	39.7 (2.3)	41.7 (2.5)	1.9 (1.6)	
		50	MA	32.6 (2.5)	32.9 (2.3)	0.4 (1.4)	
	MV-delay and MA-delay group (N = 14)	150	MV	30.8 (1.5)	37.6 (2.4)	6.9** (2.1)	4.9*
		150	MA	29.8 (1.6)	33.7 (1.9)	3.9** (1.3)	3.6*
Exp 2	MV-sync and MA-delay group (N = 14)	50	MV	29.9 (2.0)	33.8 (2.4)	3.9** (1.5)	
		150	MA	24.7 (1.7)	29.5 (1.9)	4.8** (1.3)	2.4
	MV-delay and MA-sync group (N = 14)	150	MV	30.8 (1.4)	35.5 (2.1)	4.7** (1.4)	0.7
		50	MA	27.2 (1.7)	29.6 (1.7)	2.4* (1.1)	

Standard errors of mean (SEMs) are shown in parentheses

* *p* < 0.05, one-tailed. ** *p* < 0.01, one-tailed

Before analysing ΔWSD, raw WSDs under the synchronous feedback conditions (MV-sync and MA-sync) were analysed to see whether there was a modality difference. They were entered into a repeated-measures ANOVA with exposure modality and test type (pre- vs. post-test) as within-subject factors. The ANOVA showed that the main effect of exposure modality was significant, *F*(1, 12) = 48.3, *p* < 0.001, while the other effects were not (all *p*’s > 0.05), indicating that tapping with auditory pacers yielded more stable responses (mean WSD = 32.7 ms) than tapping with visual pacers (mean WSD = 40.7 ms).

The mean ΔWSDs were entered into a mixed-model ANOVA with exposure delay (lag-50 vs. lag-150 ms) as a between-subjects factor and exposure modality (visual vs. auditory) as a within-subject factor. The ANOVA showed that the effect of exposure delay was significant, *F*(1, 25) = 4.8, *p* = 0.037, indicating that ΔWSD was significantly greater after exposure to delayed feedback (5.4 ms) than that after exposure to synchronous feedback (1.1 ms). This is shown as “ΔWSD diff per modality” in Table 2. No other effects were significant (all *p*’s > 0.05). As in the TRE, one-sample *t* tests (one sided as there was a clear prediction) on the mean ΔWSD for each condition showed that in the MV-delay and MA-delay condition, the mean MV-ΔWSD (6.9 ms) and MA-ΔWSD (3.9 ms) were significantly greater than zero, *t*(13) = 3.3, *p* = 0.003, *t*(13) = 2.9, *p* = 0.006, respectively. Variability in the MV-sync and MA-sync condition did not change (MV-ΔWSD = 1.9 ms, MA-ΔWSD = 0.4 ms, all *p*’s > 0.05).

### Discussion

The results of experiment 1 show that exposure to visual and auditory delayed feedback induced equally large TREs to auditory and visual pacers with similar build-up courses.^2^ This fits previous studies reporting equal amounts of temporal recalibration across modalities (Heron et al. 2009; Sugano et al. 2010). The bootstrap analysis, however, showed that the build-up rate for the MV-delay was somewhat faster than for the MA-delay. The results also show that the variability in timing increased after exposure to delayed feedback, regardless of modality. It is known that variability corresponds to the precision of interval discrimination (Ivry and Hazeltine 1995; Hazeltine et al. 1997; Krause et al. 2010; Merchant et al. 2013; van Vugt and Tillmann 2014). This increase in variability is therefore in line with the notion that adaptive shifts in subjective simultaneity are accompanied (or preceded) by a widening of the “window of simultaneity” (Navarra et al. 2005, 2007).

In experiment 2, we further examined whether exposure to visual and auditory delayed feedback yields identical TREs. Importantly, the apparent similarity between the MA- and MV-TRE does not imply that the same underlying mechanism is causing these effects because it is in essence a null finding. To examine this in more detail, we *mixed* in experiment 2 synchronous feedback in one modality with delayed feedback in the other modality. Participants were thus exposed to synchronous auditory feedback interleaved with delayed visual feedback, or vice versa. This allowed us to test whether adaptation to auditory or visual delayed feedback is modality specific, or rather that synchronous feedback in one modality can erase the TRE in the other modality. To be more precise, we envisaged three potential outcomes: (i) if only a general or shared component is involved in MA and MV temporal recalibration, one expects that mixing synchronous with delayed feedback will diminish the TREs in both modalities. (ii) If only sensory-specific components are involved in MA and MV temporal recalibration, one expects that the delayed modality displays a sensory-specific TRE, but not the synchronous one. (iii) A third possibility is related to the notion that audition *dominates* vision in time (Welch and Warren 1980; see Vroomen and Keetels 2010 for review), which was already evident in the smaller tapping variability in MA than in MV (Table 2). In this view, it is the timing of the auditory feedback that determines both the MA- and MV-TRE: delayed auditory feedback (combined with synchronous visual feedback) thus induces an MA- and MV-TRE, but delayed visual feedback (combined with synchronous auditory feedback) does not induce a TRE in the auditory and visual modality.

## Experiment 2: Exposure to mixed MA- or MV-delays

Participants in experiment 2 were exposed to different feedback delays in the auditory and visual modality (i.e. MV-sync and MA-delay, or MV-delay and MA-sync). The critical question was whether this mixing of feedback delays across modalities would affect the MA- and MV-TREs in the same way.

### Methods

#### Participants

Twenty-eight students from Kyushu Sangyo University participated in the experiment (two were female, mean age was 21.5, and two were left-handed but used a computer mouse by the right hand). Half of them were assigned to the MV-sync and MA-delay condition (see “Design and procedure”), the other half were assigned to the MV-delay and MA-sync condition.

#### Design and procedure

Design and procedures were same as experiment 1, except that during the exposure phase feedback delays alternated across trials so that auditory feedback was consistently delayed (or synced) and visual feedback was consistently synced (or delayed) relative to the finger taps.

### Results and discussion

#### Mean tap-asynchronies

Data were pre-processed as before. Mean tap-asynchronies and TREs are shown in Table 1. The TREs were entered into a mixed-model ANOVA with exposure delay (lag-50 vs. lag-150 ms) as a within-subject factor and modality–delay combination (MV-sync and MA-delay vs. MV-delay and MA-sync) as a between-subjects factor. The ANOVA showed an interaction between exposure delay and modality–delay combination, *F*(1, 26) = 5.1, *p* = 0.032, but there were no significant main effects (both *p*’s > 0.05). Subsequent ANOVAs per modality–delay combination with exposure delay as a within-subject factor revealed that the effect of exposure delay on TREs was significant only if sounds were delayed (the MV-sync and MA-delay condition), *F*(1, 13) = 8.4, *p* = 0.013: the size of the MA-TRE in the MV-sync and MA-delay condition (−27.6 ms) was significantly greater than that of the MV-TRE in the same condition (−14.6 ms). There was no effect of exposure delay on TREs if sounds were synchronized (the MV-delay and MA-sync condition), *F*(1, 13) = 0.1, *p* = 0.726: the size of the MA-TRE under the MV-delay and MA-sync condition (−8.1 ms) and of the MV-TRE under the same condition (−6.4 ms) were in this case not significantly different from each other.

One-sample *t* tests on the TRE for each condition showed that MV-TRE was significantly less than zero in the MV-sync and MA-delay condition (−14.6 ms), *t*(13) = 2.6, *p* = 0.012, and that MA-TRE was significantly less than zero in the MV-delay and MA-sync condition (−8.1 ms), *t*(13) = 2.1, *p* = 0.029, and in the MV-sync and MA-delay condition (−27.6 ms), *t*(13) = 3.6, *p* = 0.002. However, the MV-TRE in the MV-delay and MA-sync condition was not significantly less than zero (−6.4 ms), *t*(13) = 1.3, *p* = 0.116. Two-sample *t* tests (one sided) on the TRE difference between delay and sync per modality (shown as “TRE diff per modality” in Table 1) revealed that it was significant for the MA (−19.5 ms), *t*(26) = 2.3, *p* = 0.016, but not for the MV (8.2 ms), *t*(26) = −1.1, *p* = 0.853. With mixed exposure delays, we thus found modality-specific effects on MA- and MV-TREs: synchronized auditory feedback abolished the MV-TRE, but synchronized visual feedback did not affect the MA-TRE.

#### Build-up of TRE

Figure 3 shows a build-up of the TRE across trial blocks, together with a fitted exponential decay function. The TRE with MA-delay already became more negative than the TRE under the MV-sync in the first trial block, and this difference remained almost constant across all blocks.

**Fig. 5:**
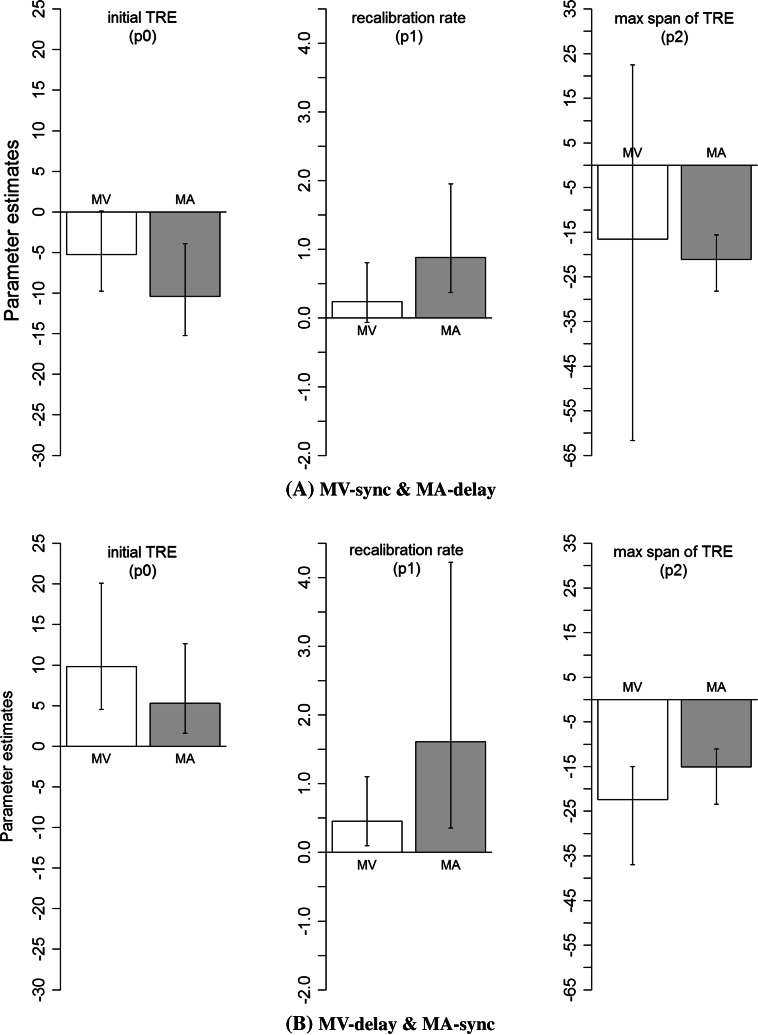
Estimated values of the parameters (p0, p1, and p2) in the nonlinear fitting for the build-up of the mean TRE in experiment 2 (exposure to mixed MV- or MA-delays): **a** MV-sync and MA-delay and **b** MV-delay and MA-sync. *Error bar* represents 95 % bootstrap confidence interval after 2000 simulations

Figure 5 shows estimated values of the parameters and 95 % bootstrap confidence intervals (CI) after 2000 simulations. As shown in Fig. 5, all the 95 % CIs are overlapped between MV and MA, meaning that they are equivalent between MV and MA.

#### Variability of tap-asynchronies

The mean within-trial standard deviation (WSD) of tap-asynchrony and the mean ΔWSD are shown in Table 2. The mean ΔWSDs were entered into a mixed-model ANOVA with exposure delay (lag-50 vs. lag-150 ms) as a within-subject factor and modality–delay combination (MV-sync and MA-delay vs. MV-delay and MA-sync) as a between-subjects factor. The ANOVA showed no significant effects of any factor (all *p*’s > 0.05).

One-sample *t* tests on the mean ΔWSD for each condition showed that it was significantly greater than zero in all conditions, MV-ΔWSD in the MV-sync and MA-delay (3.9 ms), *t*(13) = 2.7, *p* = 0.009, MA-ΔWSD in the MV-sync and MA-delay (4.8 ms), *t*(13) = 3.7, *p* = 0.001, MV-ΔWSD in the MV-delay and MA-sync (4.7 ms), *t*(13) = 3.2, *p* = 0.003, and MA-ΔWSD in the MV-delay and MA-sync (2.4 ms), *t*(13) = 2.2, *p* = 0.023. Two-sample *t* tests (one sided) on the ΔWSD difference between delay and sync per modality (shown as “ΔWSD diff per modality” in Table 2) revealed that neither MV (0.7 ms) nor MA (2.4 ms) was significant, *t*(26) = 0.4, *p* = 0.359 and *t*(26) = 1.4, *p* = 0.081, respectively.

## General discussion

The subjective timing between an action and the consequences of that action is flexible and can be recalibrated. We examined whether temporal recalibration is different for the auditory and visual modality by interleaving similar (experiment 1) or different delays (experiment 2) across modalities to find out whether the size and the build-up of MV- and MA-TRE would differ. Similarity would suggest that there is a single amodal mechanism, like a movement-related shift of timing, which underlies the sensory-motor TRE. Participants in experiment 1 made voluntary finger taps followed by either synchronous or delayed auditory and visual feedback. After a short exposure phase, they then tried to tap in synchrony with auditory and visual pacers. Temporal recalibration manifested itself in that after exposure to delayed feedback, participants compensated for this delay by tapping earlier to auditory and visual pacers. The size and the build-up of this MA- and MV-TRE were very similar to each other, confirming previous reports (e.g. Heron et al. 2009; Sugano et al. 2010). However, when the timing of the feedback signals (synchronous or delayed) was mixed across modalities (experiment 2), we found a very different pattern. Most importantly, timing of the *auditory* feedback now drove the auditory and visual TREs, but timing of the visual feedback had no effect.

How to account for this finding? One possibility is that exposure to delayed visual feedback simply induces a weak and fragile MV-TRE that is vulnerable to erasure. This seems unlikely, however, because the MV-TRE was very much like the MA-TRE in experiment 1. Moreover, earlier studies have shown that exposure to delayed visual feedback elicits a reliable MV-TRE in TOJ task (Stetson et al. 2006; Heron et al. 2009; Sugano et al. 2010; Stekelenburg et al. 2011, Tsujita and Ichikawa 2012) and in a tapping task (Sugano et al. 2012; Rohde et al. 2014b), thus arguing against the possibility that delayed visual feedback just cannot induce an MV-TRE.

More likely is that auditory feedback is, from a relative point of view, more potent than visual feedback to drive the MA- and MV-TRE. This *dominance* of audition over vision might explain why synchronized auditory feedback overrides the effect of a visual delay. The notion of auditory dominance is related to the fact that timing in audition is inherently more salient and has better temporal resolution than in vision. This was manifested in the present study in the WSD, as the WSD was larger in MV than in MA (Table 2), thus supporting a higher temporal resolution in audition than in vision (see also Grondin 1993, 2010; Grondin et al. 1998).

The results can also be viewed from the modality appropriateness hypothesis in which the more precise modality dominates the less precise modality (Welch and Warren 1980; Shimojo et al. 2001; Ernst and Bülthoff 2004; Burge et al. 2010; see Vroomen and Keetels 2010 for review). Accordingly, vision should be influenced when combined with audition, but not vice versa. A coarse temporal resolution in vision thus might prevent an adjustment of perceived visual timing because the motor-visual delay is not attributed to a consistent lag that needs to be adjusted, but rather to the inherent instability of the visual system itself. In line with this argument, it has been suggested that inaccurate perception of timing sometimes prevents temporal recalibration to occur (Vercillo et al. 2014).

It should be noted, however, that others have argued that audition should in fact be the less trusted modality because audition cannot always be relied upon, especially for distant events (Navarra et al. 2009). In this case, vision is a more “valid” (veridical) estimate of timing about a distal event than audition, despite that audition is more “reliable” (less variable) than vision. A particular example is the lightning and thunder in which the timing of lightning conveys better information about when the event actually occurred rather than the thunder that lags behind. In line with this idea, Navarra et al. (2009) reported that after exposure of AV asynchrony, the perceptual latency of the auditory modality was adapted rather than the visual one. Of note, however, is that Di Luca et al. (2009) investigated to what extent an auditory-visual (AV) temporal recalibration transfers to visual-tactile (VT) and audio-tactile (AT) pairings. They reported that exposure to an AV asynchrony induced a change in the perceptual latency of the auditory signal if the sounds were presented via headphones, but not if sounds were presented via speakers, because then visual timing changed. Presumably, then, it is the relative trust of the auditory and visual timing that is key for which modality adapts (Di Luca et al. 2009).

### Change of tapping variability with the TRE

In experiment 1, we found that not only the average tap-asynchrony, but also the variability of tapping increased after exposure to delayed feedback, while variability did not change if participants were exposed to subjectively synchronous feedback. This raises the question whether an increment of tapping variability truly reflects loss of temporal sensitivity between one’s action and its sensory feedback. It has been shown that delayed auditory feedback (DAF) disrupts motor activity utterance (stutter) and even tapping (Pfordresher and Benitez 2007; Pfordresher and Bella 2011). It is thus possible that delayed auditory feedback merely interferes with a smooth movement but does not reduce temporal sensitivity. Loss of sensitivity after exposure to asynchronous cross-sensory stimuli has been reported for audio-visual speech (Navarra et al. 2005; Vatakis et al. 2008), visuo-tactile stimuli (Navarra et al. 2007), and tactile–tactile stimuli (Winter et al. 2008). These studies using TOJ or SJ task reported that JND increased with or without a change of PSS. However, others have reported that sensitivity for cross-sensory temporal relationships (i.e. JND) does not change even after exposure to temporal asynchrony, which was accompanied with a change of PSS (Hanson et al. 2008; Harrar and Harris 2008; Sugano et al. 2010). Thus, effects of cross-sensory temporal recalibration on JND and PSS seems to be independent and do not usually covary.

Another potentially relevant factor is related to the notion of “intentional binding” (Haggard et al. 2002). Basically, it implies that the timing of a voluntary action and the sensory feedback following that action attract each other in time. Intentional binding may widen the window of simultaneity so that temporal sensitivity is decreased. Lowered sensitivity due to intentional binding might also occur in the present experiment. Of note, however, is that intentional binding cannot be the whole explanation, because earlier studies did not find an increment of tapping variability after exposure to delayed feedback (e.g. Sugano et al. 2012, 2014). One possible factor for this discrepancy might be the duration of the exposure phase. Earlier studies (Sugano et al. 2012, 2014) had relatively short exposure delays (30 or 20 trials) compared with the present study (40 trials). A longer exposure phase thus might boost the effect of a delayed feedback and induce an increment in tapping variability.

Another possibility is that auditory feedback, relative to visual feedback, actually *decreased* tapping variability and thus *stabilized* tapping. Support of this explanation comes from Harrar and Harris (2008) who reported that exposure to staggered *sound*/touch pair *increased* temporal sensitivity (decrement of JND) for subsequent *sound*/light pairs. Such a beneficial effect might come from the high temporal resolution of auditory signal, which works in the present situation as well.

### Concluding remarks

To sum up, the main finding of the present study is that exposure to a mixture of delayed/synchronous auditory/visual feedback yielded different result in synchronous tapping to flashes or beeps: an exposure to auditory delayed feedback had a dominant role that drove the MA- and MV-TREs. Visual delayed feedback had little effect, if any, when it was interleaved with auditory synchronous feedback. Presumably, this occurs because timing in audition is inherently more precise than in vision. Exposure to delayed feedback also increased tapping variability, but more research is needed to clarify whether this actually reflects a loss of temporal sensitivity or rather instability of motor control.

## Footnotes

1 It should be noted that the amount of MA tap-asynchrony was about −80 ms rather than −50 ms in our previous reports (Sugano et al. 2012, 2014), which was almost the same as in the present study (see Table 1). However, we did not observe any noticeable shift of MA tap-asynchrony after 50 ms delay exposure in those studies. Therefore, we assumed that the 50-ms delay in MA properly worked as “subjectively synchronous”.

2 It is of note that the 150-ms delay produced the same amount of TRE for the MV and the MA even though the baseline of tap-asynchrony was different between the MV (~50 ms) and the MA (~90 ms). It means that the 150-ms delay works fine with both MV and MA. However, on the other hand, it also implies that a “net delay” (a difference between a physical delay and a baseline tap-asynchrony) might be different between MV (~100 ms) and MA (~60 ms). This suggests that the MA-TRE can be produced by lesser amount of delay than for the MV-TRE.
